# Evaluation of the Protective Immunity of a Novel Subunit Fusion Vaccine in a Murine Model of Systemic MRSA Infection

**DOI:** 10.1371/journal.pone.0081212

**Published:** 2013-12-04

**Authors:** Qian-Fei Zuo, Liu-Yang Yang, Qiang Feng, Dong-Shui Lu, Yan-Dong Dong, Chang-Zhi Cai, Yi Wu, Ying Guo, Jiang Gu, Hao Zeng, Quan-Ming Zou

**Affiliations:** Department of Clinical Microbiology and Immunology, College of Medical Laboratory Science, The Third Military Medical University, Chongqing, People's Republic of China; Instituto Butantan, Brazil

## Abstract

*Staphylococcus aureus* is a common commensal organism in humans and a major cause of bacteremia and hospital acquired infection. Because of the spread of strains resistant to antibiotics, these infections are becoming more difficult to treat. Therefore, exploration of anti-staphylococcal vaccines is currently a high priority. Iron surface determinant B (IsdB) is an iron-regulated cell wall-anchored surface protein of *S. aureus*. Alpha-toxin (Hla) is a secreted cytolytic pore-forming toxin. Previous studies reported that immunization with IsdB or Hla protected animals against *S. aureus* infection. To develop a broadly protective vaccine, we constructed chimeric vaccines based on IsdB and Hla. Immunization with the chimeric bivalent vaccine induced strong antibody and T cell responses. When the protective efficacy of the chimeric bivalent vaccine was compared to that of individual proteins in a murine model of systemic *S. aureus* infection, the bivalent vaccine showed a stronger protective immune response than the individual proteins (IsdB or Hla). Based on the results presented here, the chimeric bivalent vaccine affords higher levels of protection against *S. aureus* and has potential as a more effective candidate vaccine.

## Introduction

S*taphylococcus aureus*, an opportunistic bacterial pathogen responsible for a diverse spectrum of human diseases, is the leading cause of bloodstream infections [1], endocarditis [2], lower respiratory tract infections [3], and culture-confirmed skin and soft tissue infections [4]. *S. aureus* is the most frequently isolated pathogen in hospital-associated infections [3]. The epidemiology of disease caused by *S. aureus* is strongly influenced by the rapid acquisition of antibiotic resistance, as some strains become resistant to nearly all front-line antibiotics [5]. Of particular concern is the emergence of methicillin-resistant *S. aureus* (MRSA) from community origins (CA-MRSA) and the acquisition of resistance to additional antibiotics, including vancomycin, which is often the antibiotic of last resort for CA-MRSA infections [6], [7].

Given its ability to cause life-threatening, drug-resistant infections, effective treatment for and prevention strategies against *S. aureus* infection are urgently needed. One option for controlling bacterial infections has been the introduction of vaccines.

Many virulence factors contribute to the pathogenesis of staphylococcal infections. Some of these include surface-associated adhesins, secreted toxins, iron acquisition-associated proteins and factors that enhance immune evasion [8], [9]. Are these staphylococcal virulence factors also protective antigens that enable the development of efficacious vaccines? In fact, various staphylococcal virulence factors have been identified as targets for novel therapeutics. The type 5 (CP5) and type 8 (CP8) capsular polysaccharides and Poly-*N*-acetyl-b-1-6-glucosamines (PNAG) were shown to induce protective immune responses against *S. aureus* [10]–[13]. A number of microbial surface components recognizing adhesive matrix molecules (MSCRAMMs) such as iron-responsive surface determinants A & H [14], iron-responsive surface determinant B [15], clumping factor A [16], [17], clumping factor B [18], serine aspartate repeat proteins D & E [19], collagen adhesin [20], fibronectin binding protein and Protein A [21], [22] have been tested in *in vivo* animal models and generate partially protective immune responses against *S. aureus* challenge. Alpha-toxin is a cytolytic pore-forming toxin and is one of the most potent bacterial toxins known [23], [24]. Mice immunized with an inactive form of alpha-toxin showed reduced mortality after challenge with *S. aureus* in a murine pneumonia model [25].

The concept of developing a vaccine based on multivalent antigens has been popularized in recent years [26]. The purported benefit of multivalent antigens has previously been described as targeting multiple virulence factors of pathogens that often utilize numerous virulence factors to cause disease, and the inclusion of multiple staphylococcal antigens would likely result in a more effective vaccine.

Both humoral and cellular immunity play important roles in host defense against *S. aureus* infection. Ideally, anti-staphylococcal vaccines should contain secreted as well as cell wall-associated antigens [27]. The evoked immune responses should lead to the production of antibodies and T cells producing IFN-γ and/or IL-17 [26], [28], [29], the latter being important for the mobilization and activation of neutrophils.

In this study, we constructed bivalent vaccines based on iron-responsive surface determinant B and alpha-toxin. We compared the protective efficacy of the bivalent vaccine to that of the individual proteins in a murine model of systemic *S. aureus* infection. The bivalent vaccine showed a stronger protective immunity than the individual proteins, and this protection correlated with neutralizing antibodies against alpha-toxin, opsonic antibodies specific for *S. aureus* IsdB, and both IL-17A- and IFN-γ-producing memory T cells.

## Materials and Methods

### Ethics Statement

All of the animal experiments were approved by the Animal Ethical and Experimental Committee of the Third Military Medical University (chong qing; permit number 2011-04). All surgery was performed under sodium pentobarbital anesthesia, and all efforts were made to minimize suffering. PMNs were prepared from fresh human blood collected from healthy adult volunteers. The study involving blood specimens of subjects (healthy adult volunteers) was conducted with the approval of the Ethics Review Board at Third Military Medical University and all healthy adult volunteers gave their written informed consent.

### Bacterial strains and culture conditions

*S. aureus* strain MRSA252 was obtained from the American Type Culture Collection (Manassas, VA, USA) and was used for recombinant proteins and the murine sepsis model. The bacteria were grown in tryptic soy broth at 37°C for 6 h, centrifuged at 5000g for 5 min, and subsequently washed with sterile phosphate-buffered saline (PBS). The washed bacteria were diluted with PBS to an appropriate cell concentration as determined by spectrophotometry at 600 nm.

### Cloning and expression of recombinant proteins

Genomic DNA extracted from *S. aureus* strain MRSA252 was used as the PCR template. The *isdB* gene was amplified using the forward primer 5′-CGCGGATCCATGAATGGCGAAGCAAAAGCAGC-3′ and the reverse primer 5′-TTTTCCTTTTGCGGCCGCCTATGTCATATCTTTATTAGATTCTTC-3′. The *hla* gene was amplified using the forward primer PHLAF: GCGGATCCGATTCTGATATTAATATTAAAACC and the reverse primer PHLAR: TAAGCGGCCGCTTATCAATTTGTCATTTCTTC. The *hla* gene was further mutated to hla (H35L) using the primers PH35LF: CTGAAAAAAGTATTTTATAGTTTTATC and PH35LR: CATGCCATTTTCTTTATCATAAGTGAC. For the Hla(H35L)-GGGGS-IsdB (N2 domain) construction, the first round of PCR was performed using the primers PHIF1: GCGGATCCGATTCTGATATTAATATTAAAACC and PHIR1: TAAATCGGTCATTTTGCTGCCACCGCCACCATTTGTCATTTCTTC to generate Hla(H35L)-GGGGS- using the primers PHIF2: GAAGAAATGACAAATGGTGGCGGTGGCAGCAAAATGACCGATTTA and PHIR2: TAAGCGGCCGCTTATCAATTGGCTTTAGTAAA to generate –GGGGS-IsdB(N2 domain); by employing the first round PCR products as the templates, the second round PCR was performed using the primers PHIF1: GCGGATCCGATTCTGATATTAATATTAAAACC and PHIR2: TAAGCGGCCGCTTATCAATTGGCTTTAGTAAA. Using the same method, the IsdB (N2 domain) -GGGGS- Hla (H35L) was generated with the primers PIHF1: GCGGA TCCAAAATGACCGATTTACAAGATAC, PIHR1: TTAATATCAGAATCTGCGCTGCCACCGCCACCATTGGCTTTAGTAAATG, PIHF2: CATTTACTAAAGCCAATGGTGGCGGTGGCAGCGCAGATTCTGATATTAA and PIHR2: TAAGCGGCCGCTTATCAATTTGTCATTTCTTC. For all of the amplified genes, BamHI and NotI sites were introduced at the beginning and end of the PCR products by primers. Double digested PCR products were ligated into pGEX-6P-2 and transformed with the *Escherichia coli* Xl/blue strain. The correct nucleotide sequences were confirmed at TaKaRa Inc. (Dalian, China). The resulting constructs were transformed into *Escherichia coli* strain BL21(DE3) for isopropyl-β-D-1-thiogalactopyranoside (IPTG)-induced expression according to the manufacturer's instructions and were expressed as GST fusion proteins.

### Purification of recombinant proteins and removal and detection of endotoxin

GST-tagged proteins were affinity-purified from cleared lysates with glutathione-Sepharose. Then the recombinant proteins were purified by Capto™ MMC. The protein eluate was subjected to endotoxin removal by Triton X-114 phase separation. Briefly, Mix up 1% Triton X-114 with recombinant protein eluate at 0°C for 5 min to ensure a homogenous solution, and incubate at 37°C for 5 min to allow two phases to form. Observe the lamination and harvest the supernatant by centrifugation. Remove the residual Triton X-114 by gel filtration and dialysis respectively. The endotoxin content, before and after removal, was detected by tachyplens ameboyto lysate assay (Houshiji cod Inc., Xiamen, China). The endotoxin content of recombinant proteins concentrate was reduced to less than 2.5 pg/µg after the third extraction by Triton X-114.

### Immunization and challenge infection

**Figure 1:**
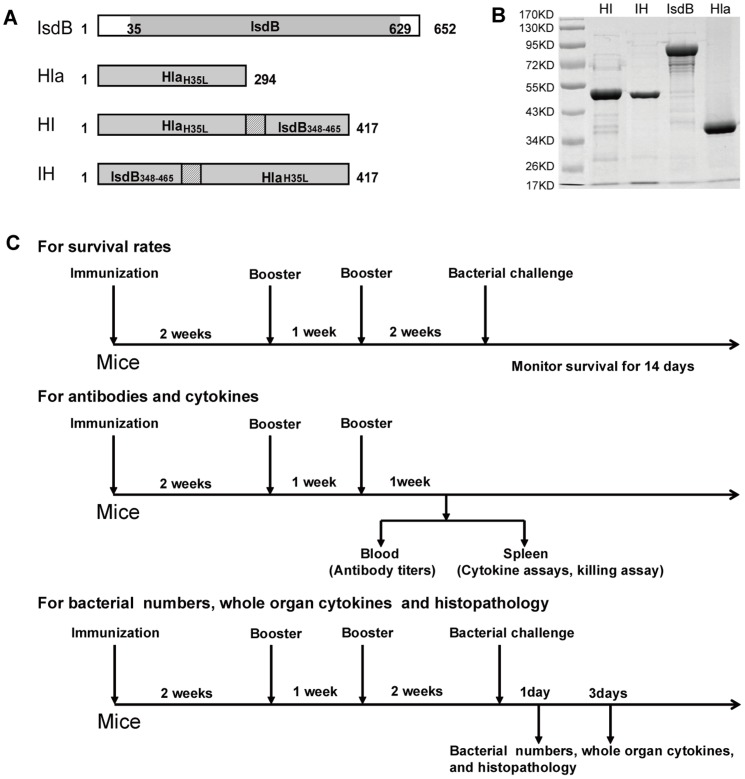
Cloning and expression of recombinant proteins as the candidate antigens of MRSA. (A) Schematic diagram illustrating the primary structure of the IsdB, Hla, HI and IH domains. (B) Recombinant GST-tagged IsdB, Hla, HI, and IH were purified by affinity chromatography and analyzed by SDS-PAGE. (C) Diagrams showing the experimental design of the immunization schedule for the measurement of the survival rates up to 14 days after bacterial challenge, for the measurement of antibodies and cytokines 1 week after the last booster and for the measurement of the bacterial numbers, whole cytokines and histopathology on days 1 and 3 after bacterial infection.

Male BALB/c mice, 6–8 weeks of age, were injected intramuscularly twice with 50 µl of the emulsion containing 20 µg of protein or with PBS plus adjuvant alum (Pierce) as a control on days 0, 14, and 21. The mice were bled on day 28, and the sera were screened by an enzyme-linked immunosorbent assay (ELISA). To determine the survival rates after *S. aureus* infection, BALB/c mice were anesthetized with sodium pentobarbital before injection and were infected with MRSA252 (1×10^9^ CFU per mouse) on day 35. The survival rates were monitored for 14 days after infection. The condition of the mice were monitored and recorded at 8, 16, and 24 o'clock every day. On the fifteenth day post infection, mice were killed by CO_2_ asphyxiation. In the survival study, although the animals died as a direct result of the intervention, our research design included plans to consider humane euthanasia for mice that were observed to be suffering severe disease or became moribund during the 14 day survival study. In detail, all animals in the survival study were sacrificed by CO_2_ asphyxiation when they became moribund as defined by a combination of ruffled fur, hunched back and dulled response to stimuli, such as finger probing, when we monitored and recorded the condition of the mice at 8, 16, and 24 o'clock every day. At the completion of all experiments, survivors were sacrificed by CO_2_ overdose in accordance with IACUC policy. To determine the bacterial numbers, whole organ cytokine concentrations and histopathology, BALB/c mice were infected with 2.5×10^8^ of *S. aureus* strain MRSA252, and the target tissues were assessed for bacterial colonization, cytokine production and histopathology on day 14 after the last booster immunization (as shown in Figure 1C).

### ELISA for specific antibodies

The mice were bled on day 7 after the last booster immunization, and their sera were screened by an ELISA [15]. The serum samples were added as the primary antibodies and the appropriate HRP-conjugated goat anti-mouse IgG antibodies, anti-IgG1-HRP or anti-IgG2a-HRP (Jackson Immuno-Research Laboratories Inc. West Grove, PA) were added as the secondary antibodies. The titer of each antibody was defined as the highest dilution giving an absorbance value of more than twice that of the blank control.

### Assess opsonophagocytic killing and neutralizing activity of antisera

Rabbits immunized with *S. aureus* antigens were tested for functional activity in a classic *in vitro* opsonophagocytic killing assay. Briefly, PMNs were isolated from healthy adult human volunteers, washed, counted, examined for viability by trypan blue exclusion, and diluted to 1–2×10^6^ PMNs per ml. cross-reactive antibodies in infant rabbit serum were removed by incubation with suspensions of *S. aureus* MRSA252 by mixing at 4°C for 30 min. Serum was then centrifuged, filter-sterilized, and used as a source of complement. *S. aureus* MRSA252 was adjusted to 1–2×10^5^ CFU per ml. Equal volumes (100 µl) of PMNs, complement, bacteria, and diluted antibodies were mixed and incubated at 37°C for 90 min prior to dilution, agar plating, and bacterial enumeration. Bacterial killing was calculated as the percent difference in CFU between samples without or with PMNs. Functional antibodies to alpha toxin neutralize the pore-forming capacity of this toxin. Hemolysis of rabbit erythrocytes is the easiest way to measure alpha toxin activity. Serial two-fold dilutions of the Hla antiserum were incubated with 4 hemolytic units of wild-type alpha toxin (Toxin Technology) for 30 min at room temperature. An equal volume of washed 1% rabbit erythrocytes will be added, and the incubation will be continued for another 30 min at 37°C. The inverse of the dilution at which no hemolysis is detectable at 545 nm will be recorded as the neutralizing titer.

### Splenic lymphocyte priming and response

The lymphocyte cytokine response was analyzed based on a modification of a previously described method [17]. The spleen cells were suspended at a concentration of 2×10^6^ cells/ml in RPMI 1640 medium supplemented with 10% fetal calf serum (FBS, HyClone Laboratories). The spleen cells were incubated at 37°C with or without 5 µg/ml of recombinant proteins. The supernatants were collected after 5 days of incubation, and the samples were stored at −80°C until further analyses were performed.

### Cytokine assay

The amounts of IFN-γ, IL-5, and IL-17A in the supernatants were determined by ELISAs using a mouse IFN-γ valukine™ ELISA kit, mouse IL-5 valukine™ ELISA kit, and IL-17A valukine™ ELISA kit (R&D Systems), respectively.

### Killing assay

The *S. aureus* killing assay was modified based on a previously described assay [30]. PMNs were harvested from the peritoneum and adjusted to 2×10^6^/ml in RPMI 1640 plus 10% fetal bovine serum. Neutrophil monolayers were incubated for 4 hours in 10% conditioned media (from vaccinated or control splenic lymphocytes exposed to recombinant proteins for 5 days) plus 90% complete media. The conditioned media were then aspirated, and the microorganisms were added to the PMNs at an MOI of 0.1. The cells and bacteria were mixed and incubated at 37°C for 90 min, and the bacteria were plated on 4% blood heart infusion (BHI) agar for bacterial enumeration. The bacterial killing was calculated as the percent reduction in the CFU in wells containing co-cultures of phagocytic cells and microorganisms compared to wells containing microorganisms alone.

### Bacterial burden, organ cytokines and histopathology

On days 1 and 3 post infection, the blood, spleens, and kidneys were harvested for the determination of the bacterial burden. The bacterial numbers in the organs were enumerated by preparing organ homogenates in PBS and plating 10-fold serial dilutions on tryptic soy agar (BD Diagnosis System). The colonies were counted after 24 h of incubation at 37°C. Whole organ cytokines were analyzed from kidneys homogenized with protease inhibitors (pepstatin, leupeptin, and PMSF) by ELISA (R&D Systems) per the manufacturer's instructions. For histopathology, the organs were fixed with 10% phosphate buffered formalin and embedded in paraffin. Four-micrometer thick sections were prepared and stained with hematoxylin and eosin for microscopic examination.

### Statistical analysis

The non-parametric log rank test was utilized to determine differences in the survival times. The Mann-Whitney U test was used to compare bacterial burden and cytokines levels. Analyses were performed using GraphPad Prism 5.0 (GraphPad Software). *P*<0.05 was considered significant.

## Results

### Immunization with a chimeric bivalent antigen induced the strongest immunity against lethal challenge with *S. aureus* MRSA252

As shown in Figure 1A, the optimized *isdB*, *hlA*, HI (Hla_H35L_-GGGGS- IsdB_348-465_), and IH (IsdB348-465 -GGGGS- Hla_H35L_) genes were amplified by PCR. All recombinant fragments were cloned into the pGEX-6P-2 vector. Following IPTG induction, the recombinant fragments were expressed as soluble proteins. The purification of the recombinant proteins from *Escherichia coli* strain BL21 (DE3) cells was efficient. The recombinant proteins corresponded to their predicted molecular masses by SDS-PAGE (Figure 1B).

**Figure 2:**
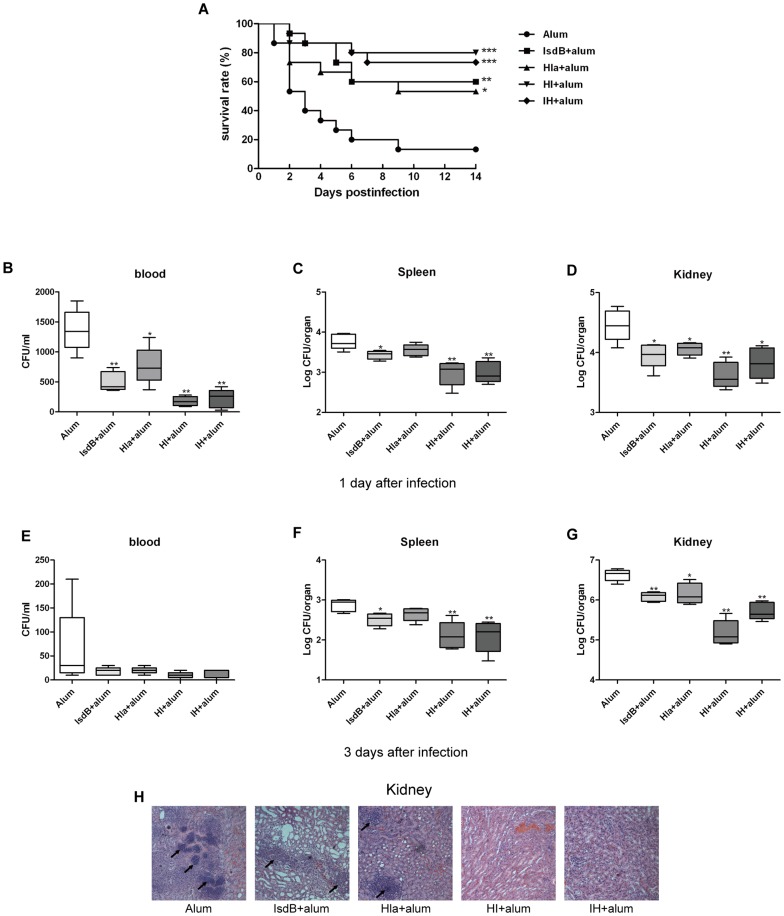
Immunization with the recombinant protein vaccine (IsdB, Hla, HI, and IH) generates protective immunity against MRSA252 challenge. (A) BALB/c mice (n = 15) were immunized with individual antigens (IsdB, Hla, HI, IH) and alum adjuvant. The animals were challenged by intravenous injection of MRSA252 (1×10^9^ CFU) and were monitored for 14 days. Compared with animals receiving antigen-free PBS and the adjuvant alone, the significance of the protective immunity generated by the various antigens was measured with a log rank test: IsdB, *P* = 0.0023;Hla, *P* = 0.0135; HI, *P* = 0.0002; IH, *P* = 0.0003. The asterisks represent a statistically significant difference (*P<0.05, **P<0.01, ***P<0.001). Representative results from one of three independent experiments are shown. Bacterial numbers in the blood, spleens and kidneys of immunized and control mice were determined at 1 (B to D) and 3 days (E to G) after infection with 2.5×10^8^ CFU i.v. Each group included 5 mice. Data are presented as box plots, and the medians and interquartile ranges are shown. Asterisks indicate significant differences between vaccinated and control mice (* *P*<0.05, ** *P*<0.01). (H) Hematoxylin-eosin-stained kidneys from mice immunized with recombinant proteins. BALB/c mice were immunized with individual recombinant proteins or injected with antigen-free PBS and alum adjuvant. Two weeks after the last booster, the mice were infected with *S. aureus* MRSA252 at 2.5×10^8^ CFU. Three days after infection, the kidneys were harvested and stained. Representative histopathological sections from 5 mice per group are shown (magnification = 100×). Arrows indicate Staphylococcal abscesses.

The mice were immunized with the bivalent HI or IH vaccines or with individual antigens alone (IsdB or Hla_H35L_) three times at one- to two-week intervals. Fourteen days after the last immunization, the mice were infected via the tail vein with 1×10^9^ cells of *S. aureus* MRSA252. The animals vaccinated with the IsdB, Hla_H35L_, HI or IH antigens all displayed higher survival rates (60%, 53.3%, 80% and 73.3% at 14 days, respectively) than the alum adjuvant control group (13.3% survival). The significance of protective immunity generated by the various antigens was measured with a log rank test (IsdB, *P* = 0.0023; Hla, *P* = 0.0135; HI, *P* = 0.0002; IH, *P* = 0.0003). The mice immunized with the bivalent HI vaccine exhibited survival rates that were 6.7%, 20%, 26.7% and 66.7% higher than the mice immunized with IH, IsdB, Hla, and adjuvant, respectively (Figure 2A). These results suggest that immunization with a recombinant bivalent vaccine can generate increased protection against lethal challenge with *S. aureus* MRSA252.

### Immunization with vaccine antigens significantly reduced both organ burden and pathology

To determine whether the recombinant vaccine protects against bacterial growth *in vivo*, the blood, spleens and kidneys from the immunized and control animals injected with the adjuvant alum were harvested and counted at days 1 and 3 after *S. aureus* MRSA252 infection (2.5×10^8^ CFU). The blood, spleens and kidneys from mice actively immunized with the recombinant vaccine had lower levels of *S. aureus* than those in the control mice immunized with the alum adjuvant (Figure 2B–G). These results suggest that the immune responses against the recombinant proteins were able to partially protect against *S. aureus* colonization. Intriguingly, in contrast to the immunization with a single vaccine antigen (IsdB, Hla_H35L_), the recombinant bivalent vaccine (HI, IH) afforded a higher level of protection against *S. aureus* challenge.

H&E-stained sections were used to investigate the histopathological alterations by light microscopy. The histological analysis showed that the kidneys of mice immunized with the recombinant vaccines had smaller renal abscesses after infection than the mice immunized with the alum adjuvant (Figure 2H). Further, the kidneys of the mice immunized with HI did not present staphylococcal abscesses. In contrast, the kidneys from mice immunized with the alum adjuvant harbored bacterial abscesses with large foci of staphylococci.

### Immunization with the chimeric bivalent antigen induced strong antibody response

**Figure 3:**
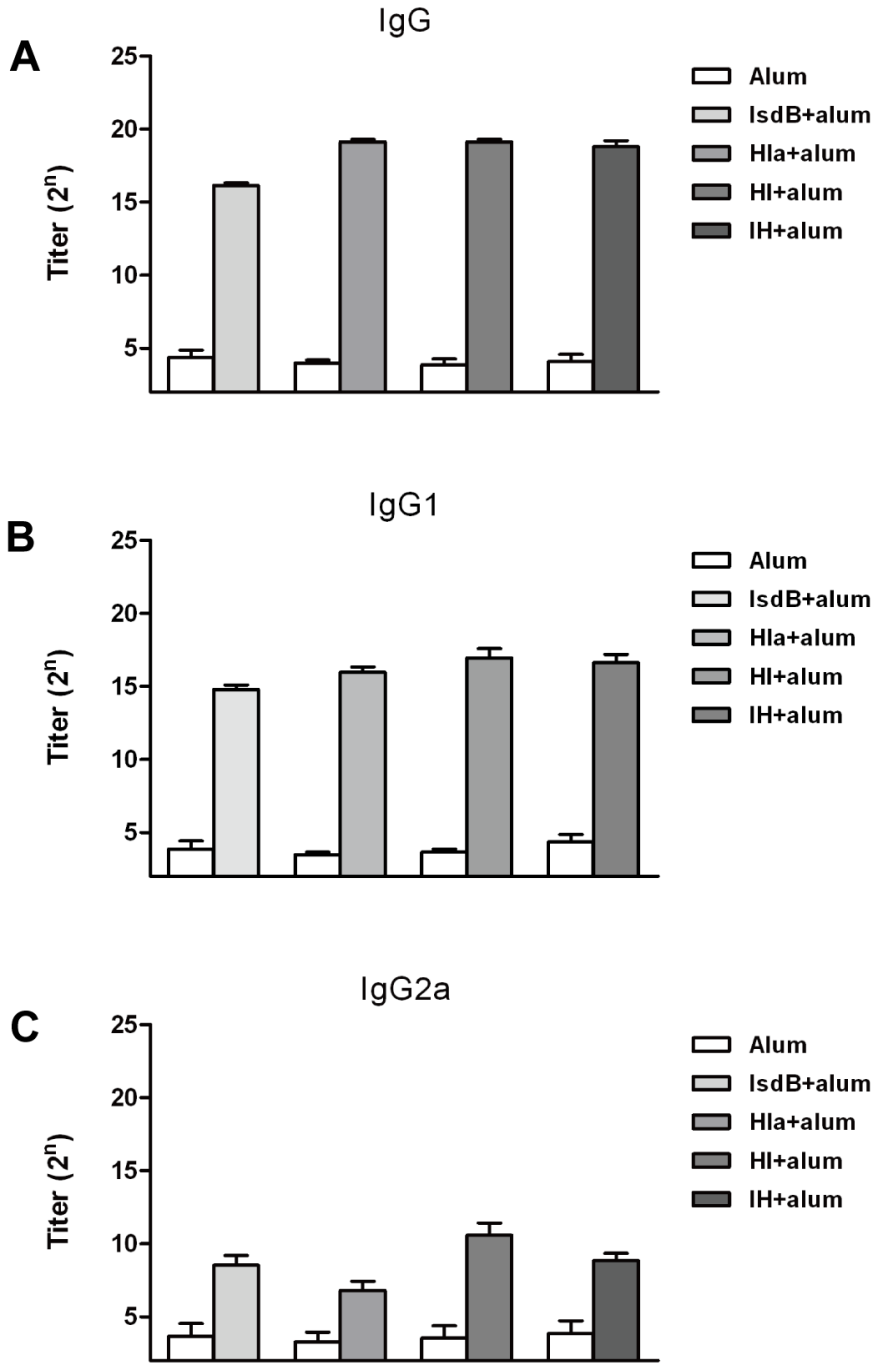
Antibody production induced by active immunization with individual recombinant proteins. BALB/c mice were immunized with recombinant proteins as described in the Materials and Methods. One week after the last booster, the mice (n = 6) were bled, and the sera were tested by ELISA for anti-recombinant protein antibodies. The titers were determined for IgG (A), IgG1 (B), and IgG2a (C). The results represent the means and standard error for a group of mice.

To evaluate the efficacy of the recombinant protein vaccine in actively immunized mice, the titers of specific antibodies against the different recombinant proteins were determined by ELISA one week after the last booster. The analysis of pooled sera from the mice immunized with recombinant proteins plus alum adjuvant indicated intense antigen-specific antibody responses. The titers of IgG against recombinant IsdB, Hla_H35L_, HI, and IH were 74667±10667, 597333±85333, 597333±85333, and 533333±116457, respectively (Figure 3). Administration of the immunizing antigens did not alter the specific antibody titers against IsdB or Hla_H35L_ (Table 1).

**Table 1:** IgG titers of specific antibodies for recombinant proteins (IsdB and Hla).

Recombinant protein	anti-IsdB antibody a	anti-Hla antibody a	significance^b^
IsdB	74670±10670	ND	
Hla	ND	597300±85330	
HI	48000±7155	853300±107900	*P = 0.0646; **P = 0.0924
IH	45330±8682	768000±114500	§ *P* = 0.0587; §§ *P* = 0.2598

a IgG titers (mean serum titers ± SEM) in response to immunization of mice as determined by ELISA (n = 6 animals).

b Specific antibody titers raised against IsdB or Hla were not significantly different following the administration of the immunizing antigens. *P, anti-IsdB antibodies from the mice immunized with IsdB were compared to those from the mice immunized with HI; **P, anti-Hla antibodies from the mice immunized with Hla were compared with those from the mice immunized with HI.

§ *P*, anti-IsdB antibodies from the mice immunized with IsdB were compared to those from the mice immunized with IH.

§§ *P*, anti-Hla antibodies from the mice immunized with Hla were compared with those from the mice immunized with IH.

### Opsonic activity of antibodies to IsdB against the *S. aureus* MRSA252 and Neutralizing activity of polyclonal anti-Hla serum

**Figure 4:**
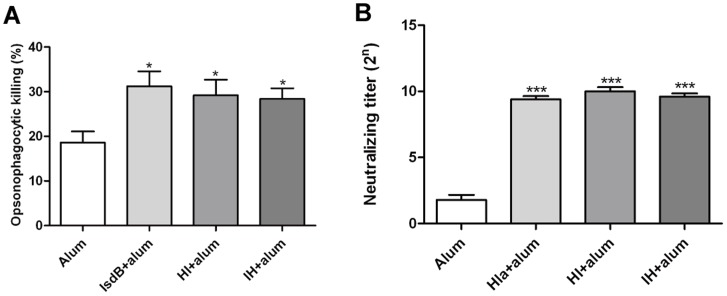
Opsonic activity of antibodies to IsdB against the *S. aureus* MRSA252 and neutralizing activity of polyclonal anti-Hla serum. (A) *S. aureus* MRSA252 (1-2×10^5^ CFU) was incubated in the presence of isolated human polymorphonuclear leukocytes (1–2×10^6^ PMNs) and diluted rabbit antisera against IsdB or normal rabbit sera in the presence of infant rabbit complement. They were plated on agar medium to measure bacterial survival as determined by CFU after 90 minute incubation. Then the percent of killing was calculated. The data shown are the means and the standard error of the means derived of 3 to 5 independent experiments. Unpaired 2 tailed student's t- tests were perfomed to analyze the statistical significance of Data comparing non-reactive rabbit anti-serum with rabbit serum raised against specific antigens IsdB (*P* = 0.0174), HI (*P* = 0.0386), and IH (*P* = 0.0219). (B) Serial two-fold dilutions of the Hla, HI, and IH antiserum (n = 5) was incubated with 4 hemolytic units of wild-type alpha toxin for 30 min at room temperature. An equal volume of washed 1% rabbit erythrocytes were added, and the incubation continued for another 30 min at 37°C. The inverse of the dilution at which no hemolysis is detectable at 545 nm was recorded as the neutralizing titer. The neutralizing titer was significantly increased for antisera from the rabbit immunized with Hla, HI, or IH compared with the antisera from the rabbit immunized with alum adjuvant only; Hla(***P<0.001), HI(***P<0.001), and IH(***P<0.001). Error bars represent the SEM.

The opsonophagocytic killing by immune cells plays an important role in host clearance the *S. aureus*. To determine the nature of protection of antibodies against IsdB, we analyzed their ability to induce opsonophagocytic killing of *S. aureus* in the presence of PMNs and complement. PMN killing of S. aureus was monitored by using a bacterial burden assay. As shown in Figure 4A, about 30% of *S. aureus* was killed by PMNs when incubated with antibodies against IsdB, HI, or IH and infant rabbit serum with complement activity, and the percents of antibody mediated staphylococci killing significantly increased when serum was used from the rabbit immunized against IsdB, HI or IH versus when antibodies were used from mock immunized rabbit. Hemolysis of rabbit erythrocytes is the easiest way to measure alpha-toxin activity in order to assess if polyclonal immune serum inhibits the hemolytic activity of alpha-toxin. Serial two-fold dilutions of the antisera were incubated with 4 hemolytic units of wild-type alpha-toxin (Toxin Technology) for 30 min at room temperature. An equal volume of washed 1% rabbit erythrocytes was added, and the incubation was continued for another 30 min at 37°C. The inverse of the dilution at which no hemolysis is detectable at 545 nm was recorded as the neutralizing titer, as shown in Figure 4B. The titers of antisera from the rabbit immunized with Hla, HI or IH, at which no hemolysis was detectable at 545 nm, were significantly increased compared with the titer of the serum from the mock rabbit. This indicated the antigens Hla, HI and IH would elicit functional antibodies to alpha-toxin that may neutralize the pore-forming capacity of this toxin.

### Immunization with the chimeric bivalent antigen induced the strongest T cell responses

**Figure 5:**
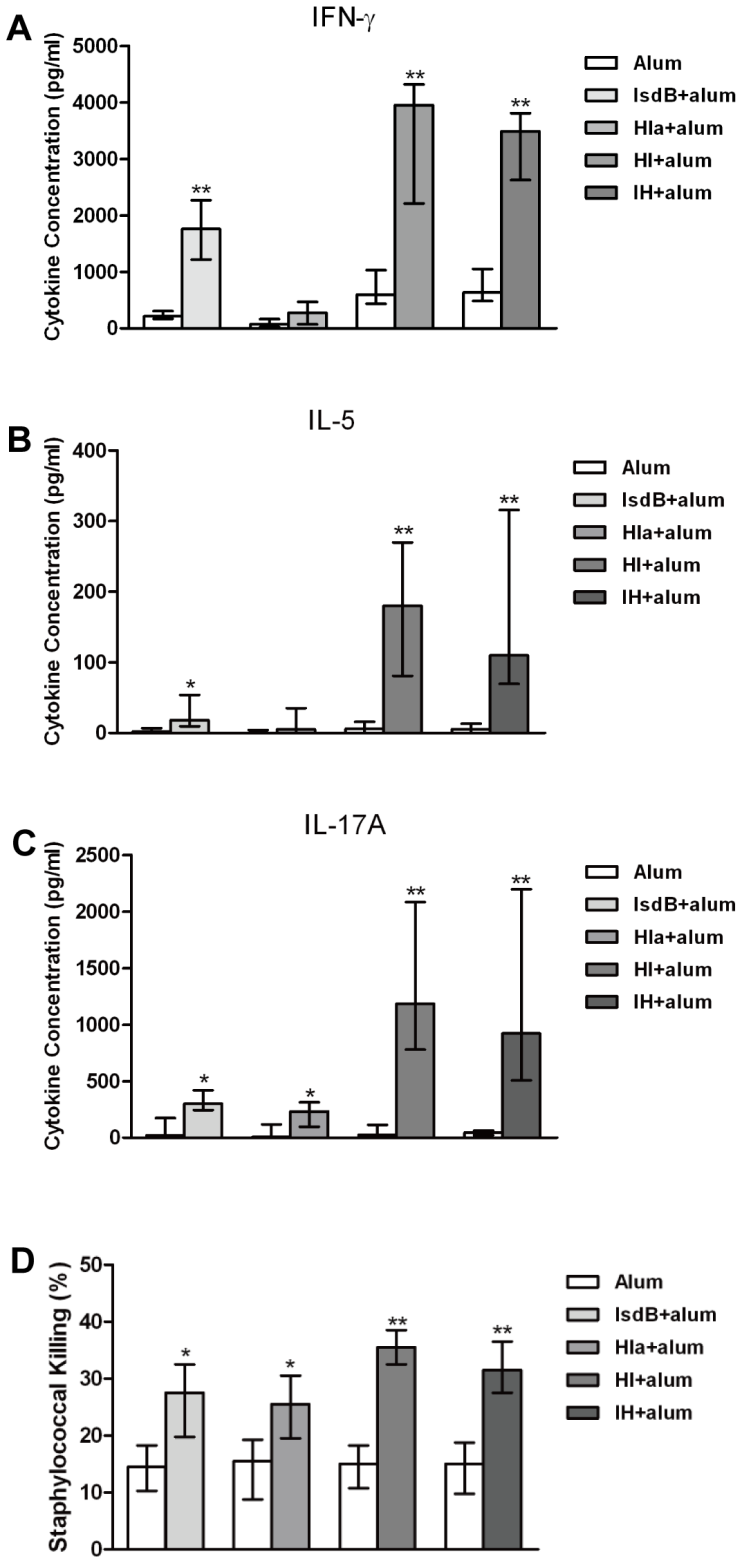
Recombinant antigen vaccines administered with alum adjuvant prime murine spleen cells for cytokine responses. One week following the last booster, spleen cells of mice (n = 6) immunized with antigen-free PBS and alum adjuvant or from mice immunized with recombinant proteins (IsdB, Hla, HI, IH) were incubated for 5 days with antigen proteins (5 µg/ml). The supernatants were harvested, and the cytokine levels of (A) IFN-γ, (B) IL-5, and (C) IL-17A were determined. The medians and interquartile ranges are shown. Asterisks indicate significant differences between the vaccinated and control mice (* *P*<0.05, ** *P*<0.01). (D) Role of cytokines in splenocyte supernatants after 5 days of co-culture with recombinant proteins in the phagocytic killing of staphylococci. PMNs were primed with supernatants for four hours, and *S. aureus* (1:10 *S. aureus* to PMNs) was added for an additional ninety minute incubation period. The bacteria were then plated on agar medium to measure the bacterial survival as CFU. The medians and interquartile ranges are shown. Asterisks indicate significant differences between the immune and control supernatants (* *P*<0.05, ** *P*<0.01).

To investigate the cytokine profiles in the spleen cells of mice immunized with recombinant proteins and control mice immunized with alum adjuvant one week after the last booster, spleen cells from all of the groups were stimulated with the recombinant proteins for 5 days, and cytokine production was analyzed in the supernatants. The splenocytes from vaccinated mice (IsdB, Hla, HI, IH) produced significantly more IFN-γ (Figure 5A) (IsdB, *P* = 0.0043; HI, *P* = 0.0087; IH, *P* = 0.0043), IL-5 (Figure 5B) (IsdB, *P* = 0.0154; HI, *P* = 0.0048; IH, *P* = 0.0048), and IL-17A (Figure 5C) (IsdB, *P* = 0.0152; Hla, *P* = 0.02; HI, *P* = 0.0022; IH, *P* = 0.0050) in response to the recombinant proteins IsdB, Hla, HI or IH than did splenocytes from control mice. Further, the splenocytes from vaccinated mice (HI, IH) produced significantly more IFN-γ (Figure 5A) (HI, *P* = 0.0411; IH, *P* = 0.0411), IL-5 (Figure 5B) (HI, *P* = 0.0087; IH, *P* = 0.0087), and IL-17A (Figure 5C) (HI, *P* = 0.0043; IH, *P* = 0.0412) in response to the recombinant proteins HI or IH than did splenocytes from control mice who had received IsdB.

### Vaccination primed lymphocytes to produce cytokines that in turn enhanced phagocytic staphylococcal killing

To determine the nature of protection, cytokines were monitored in the supernatants from cells stimulated with recombinant proteins and analyzed the supernatants for their ability to induce staphylococcal killing in the presence of PMNs, as this bacterial killing is one of the most important immunological correlates of protective immunity against *S. aureus*. The supernatants from stimulated immune cells exhibited markedly enhanced phagocytic killing of *S. aureus* ex vivo compared to supernatants from the control cells (Figure 5D). Additionally, the supernatants from immune cells stimulated with HI or IH caused an increase in phagocytosis compared to the supernatants from immune cells stimulated with IsdB or Hla.

### Inflammatory cytokine and CXC chemokine responses after systemic infection

**Figure 6:**
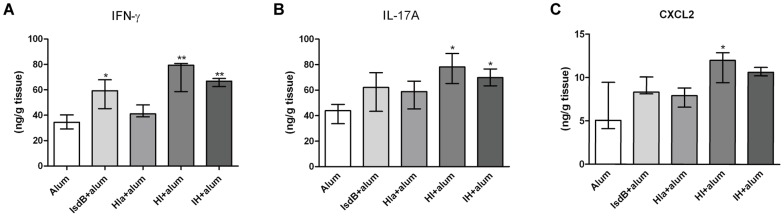
Whole organ cytokine levels in the kidneys of mice immunized with recombinant proteins. BALB/c mice were immunized with individual recombinant proteins or injected with antigen-free PBS and alum adjuvant. Two weeks after the last booster, the mice were infected with *S. aureus* MRSA252 (2.5×10^8^ CFU). Three days after infection, the kidneys of the mice were obtained and homogenized. The cytokine levels in organ homogenates were determined by ELISA. (A)IFN-γ, (B) IL-17A, and (C) CXCL2 expression in the kidneys. Each group included 5 mice, and the medians and interquartile ranges are shown. * *P*<0.05, ** *P*<0.01 vs. control by the Mann-Whitney U test.

To determine the levels of cytokines and chemokines in the kidneys of vaccinated versus control mice at days 3 after infection, the cytokines IFN-γ and IL-17A and the CXC chemokine CXCL2 were measured. The results demonstrated that the concentrations of IFN-γ, IL-17A, and CXCL2 were higher in the kidneys of vaccinated than control mice (Figure 6A-C). Further, immunization with HI or IH resulted in higher levels of IFN-γ, IL-17A, and CXCL2 than immunization with IsdB or Hla (Figure 6A–C). Lastly, immunization with HI resulted in the highest levels of IFN-γ, IL-17A, and CXCL2 among all groups.

## Discussion

*S. aureus* disease is a deeply concerning problem in healthcare as well as community settings. Screening and defining *S. aureus* antigens will be the key to future vaccine development.

IsdB is an iron-regulated cell wall-anchored surface protein of *S. aureus* that plays an important role in heme iron acquisition [31]. Previous studies independently confirmed the protective capacity of IsdB in active vaccination [15], [19], [32]. Kuklin et al demonstrated that mice immunized with recombinant IsdB showed improved survival following intravenous challenge with five of six clinical strains of *S. aureus* [15]. In a second study, immunization with four antigens (IsdA, IsdB, SdrD, and SdrE) generated significant protective immunity that correlated with the induction of opsonophagocytic antibodies [19].

Alpha-toxin is a secreted *S. aureus* protein that can cause pore formation within eukaryotic cells and interfere with *S. aureus* adhesion to epithelial cells [33]. Wardenburg and Schneewind used an inactive form of alpha-toxin to immunize mice and evaluate the protection against *S. aureus* in a murine pneumonia model. Compared to animals immunized with PBS, mice immunized with the H35L protein and then challenged intranasally with *S. aureus* showed reduced mortality [25].

In this study, we confirmed that immunization with IsdB or Hla was partially protective against *S. aureus* disease in a bacteremia infection model by evaluation of survival rates, bacterial colonization and histopathology. Survival rates increased and bacterial counts decreased in the group of mice immunized with multiple recombinant proteins. The histopathological analysis showed that there is less abscess formation in the kidneys from the mice immunized with the specific antigens compared to those immunized with the alum adjuvant alone. Moreover, vaccination with the bivalent antigens HI or IH resulted in better protection than vaccination with a single vaccine antigen. The titers of antibodies against individual components were comparable in the groups receiving a single-valency vaccine and two bivalent vaccines. We concluded that the bivalent vaccines elicited similar opsonophagocytic killing as the single-antigen IsdB vaccine and elicited neutralization activity comparable to the single-antigen Hla vaccine.

To date, multiple attempts to develop a vaccine to prevent Staphylococcus aureus infection have failed. Most of these vaccines have been based upon the development of opsonic antibodies. New information suggests that cell-mediated immunity may play a critical role in the development of adaptive immunity against *S. aureus*.

Th1 cells produce IFN-γ and activate immune cells to trigger defense against pathogens. Zhao et al investigated the effects of supplementation with and neutralization of IFN-γ in a murine model of septicemia and concluded that the beneficial effect correlated to increased phagocytosis and bacterial clearance [34]. Lin et al found that IFN-γ-deficient mice are hypersusceptible to infection caused by intravenous inoculation with *S. aureus*. IFN-γ-deficient mice receiving immune CD4+ lymphocytes from vaccinated, wild type donor mice displayed improved survival after infections. Hence, IFN-γ produced by vaccine-primed CD4+ T cells was required for mediating adaptive immunity against infections [30].

IL-17 is an important bridging molecule between the adaptive and innate immune systems, as it arises from the adaptive immune system and alerts the innate immune system to enhance inflammation and mobilize neutrophils to limit the spread of infection. Lin et al reported that a Candida adhesin vaccine protected wild-type mice against infection with *S. aureus* and that the vaccine-mediated protection required CD4+ T cell-derived IL-17. Likewise, Narita et al reported that immunization with *S. aureus* ClfA induced IL-17A-producing cells in vivo and that immunization protected wild type, but not IL-17A-deficient mice, against invasive infection [17]. Recently, Joshi et al reported that IsdB immune T cells were critical for protection of SCID mice challenged with a lethal dose of *S. aureus*. Further study showed that Th17 cells were necessary for the IsdB-generated protective immunity [35].

In our study, we confirmed that the IsdB vaccine primed the spleen cells to produce high levels of both IFN-γ and IL-17A. Moreover, the bivalent vaccine primed the spleen cells to produce higher levels of IFN-γ, IL-5, and IL-17 than a single vaccine antigen. Vaccination primed lymphocytes to produce cytokines and enhanced phagocytic staphylococcal killing. Immunization with recombinant proteins resulted in high levels of IFN-γ, IL-17A, and CXCL2. They enhance neutrophil-mediated killing of bacteria. These results suggest that recombinant vaccines can elicit cellular immunity and enhance bacterial elimination by neutrophils. In particular, the bivalent vaccines elicited stronger cell-mediated immune responses which are important for the prevention of *S. aureus* infection than a single vaccine antigen.

In summary, the recombinant antigens improved the clinical outcomes in a murine model of systemic *S. aureus* infection by inducing humoral and cellular immunity. Moreover, our data suggest that the bivalent vaccine can elicit immune responses that achieve greater protective immunity than immunization with the individual *S. aureus* components and that the two-target approach provides enhanced protection against systemic *S. aureus* infection. However, further examination is required to evaluate the humoral and cell-mediated immune responses, which are both important for an effective vaccine against *S. aureus*.
